# AT2R Activation Improves Wound Healing in a Preclinical Mouse Model

**DOI:** 10.3390/biomedicines12061238

**Published:** 2024-06-03

**Authors:** Julia M. Harrison, Edwin K. Leong, Natasha D. Osborne, Jean S. Marshall, Michael Bezuhly

**Affiliations:** 1 Department of Surgery, IWK Health Centre, 5850/5980 University Avenue, Halifax, NS B3K 6R8, Canada; julia.harrison@dal.ca; 2 Department of Surgery, Dalhousie University, 5850 College St, Halifax, NS B3H 4H7, Canada; 3 Department of Pathology, Dalhousie University, 5850 College St, Halifax, NS B3H 4H7, Canada; 4 Department of Microbiology & Immunology, Dalhousie University, 5850 College St, Halifax, NS B3H 4H7, Canada; natasha.osborne@dal.ca

**Keywords:** angiotensin II type 2 receptor, compound 21, wound healing, fibrosis, scarring

## Abstract

Abnormal skin healing resulting in chronic wounds or hypertrophic scarring remains a major healthcare burden. Here, the antifibrotic angiotensin II type 2 receptor (AT2R) signaling pathway was modulated to determine its impact on cutaneous wound healing. Balb/c mice received two splinted full-thickness wounds. Topical treatments with the selective AT2R agonist compound 21 (C21) and/or selective antagonist PD123319 or saline vehicle were administered until sacrifice on post-wounding days 7 or 10. The rate of wound re-epithelialization was accelerated by PD123319 and combination treatments. In vitro, C21 significantly reduced human fibroblast migration. C21 increased both collagen and vascular densities at days 7 and 10 post-wounding and collagen I:III ratio at day 10, while PD123319 and combination treatments decreased them. Genes associated with regeneration and repair were upregulated by C21, while PD123319 treatment increased the expression of genes associated with inflammation and immune cell chemotaxis. C21 treatment reduced wound total leukocyte and neutrophil staining densities, while PD123319 increased these and macrophage densities. Overall, AT2R activation with C21 yields wounds that mature more quickly with structural, cellular, and gene expression profiles more closely approximating unwounded skin. These findings support AT2R signal modulation as a potential therapeutic target to improve skin quality during wound healing.

## 1. Introduction

Postnatal human wounds heal with the formation of scar tissue. When the fibrotic activity in a wound is prolific or prolonged, such as after a burn injury, hypertrophic scarring can result. Hypertrophic scarring can lead to joint contractures, pain, pruritus, and reduced sensation [1,2,3,4]. Additionally, severe scarring can lead to disfigurement and profound psychological distress [5]. Current therapies to prevent or reduce severe scarring are limited and only modestly effective, with surgery frequently being the only option [1,4]. Globally, the functional and aesthetic sequelae of hypertrophic scarring pose a significant burden not only to patients but also to the health systems that care for them [2,4,5]. To address this burden, novel, noninvasive therapies to reduce scarring are needed.

Unlike loose-skinned animals like mice, whose wounds close rapidly by contraction, human skin is tethered to the underlying fascia, which resists these contractile forces. As a result, full-thickness human skin healing occurs through a series of overlapping phases ultimately leading to re-epithelialization [4]. In the inflammatory phase, tissue-resident mast cells release chemokines to recruit neutrophils that secrete proteases such as matrix metalloproteinases (MMPs) to clear cellular debris and bacteria [4,6,7]. The presence of neutrophils in a wound is typically short-lived, and when neutrophils persist in a wound, the proteases they release cause continued tissue damage consistent with chronic inflammation [4,6,8]. During the inflammatory phase, macrophages serve as a source of many pro-inflammatory and pro-fibrotic mediators, including interleukin 6 (IL6) and transforming growth factor β (TGF-β), respectively [8,9]. Both neutrophils and macrophages stimulate the proliferation of fibroblasts and keratinocytes to initiate the proliferative and re-epithelialization phases. As wound healing progresses into remodeling, macrophage activity shifts to clearing debris by phagocytosis and inhibiting the inflammatory response [6,8,10].

During the proliferative phase, fibroblasts produce extracellular matrix (ECM) proteins such as collagen type III and fibronectin [4,8,9]. Fibroblasts, macrophages, and mast cells promote angiogenesis within the wound, leading to a dense capillary bed [4,7,9,10]. As the proliferative and re-epithelialization phases end, an extended remodeling phase ensues in which the ECM proteins initially laid down during proliferation are reorganized and inflammatory cells undergo apoptosis [8,9]. Fibroblasts remodel the ECM to increase collagen density and the proportion of mature collagen type I to the immature collagen type III [11,12,13]. Strategies to improve wound healing therefore seek to reduce early inflammation, limit fibroblast activation, and promote efficient remodeling to prevent the excess accumulation of scar tissue.

One potential strategy is the modulation of angiotensin II (AngII) signaling. AngII is the end-product of the renin–angiotensin system (RAS). AngII engagement of the AngII type I receptor (AT1R) is profibrotic, and its selective blockade has been shown to inhibit fibrosis in a variety of settings [14,15,16]. Given its critical role in regulating blood pressure and water and electrolyte homeostasis, clinically targeting AT1R is problematic, as angiotensin receptor blocker (ARB) or angiotensin-converting enzyme inhibitor (ACEI) doses capable of inhibiting fibrosis also lead to hypotension [17,18,19]. Additionally, AngII can bind to the AngII type 2 receptor (AT2R), which is upregulated in areas of injury and remodeling [14]. AT2R signaling has been shown to be anti-fibrotic and proangiogenic in animal models of skeletal muscle, neurologic, and gastrointestinal diseases [14,20,21]. To this end, AT2R signaling represents a more attractive therapeutic target for preventing fibrosis.

Compound 21 (C21) is a selective AT2R agonist that has been shown to be well tolerated and efficacious in Phase 2 clinical trials to treat idiopathic pulmonary fibrosis and a Phase 3 trial to restore lung function in hospitalized COVID-19 patients [22]. In a mouse xenograft model of Dupuytren disease, we have shown that C21 reduces myofibroblast expression and TGF-β transcription [18]. Additionally, C21 reduces intra-abdominal adhesions in a mouse model [23]. Given these antifibrotic effects of AT2R engagement, we sought to investigate the role of AT2R signaling in a mouse model of cutaneous wound healing. To compare C21 treatment with the counter-regulatory action of AT2R, we utilized the selective AT2R antagonist, PD123319, which has been shown to enhance pathways associated with cellular proliferation, motility, and fibrosis [24,25,26]. Here, AT2R activation via C21 was found to improve wound maturity, with cellular and structural characteristics approximating unwounded skin, while blocking the receptor with PD123319 resulted in wounds that re-epithelialized more rapidly, but with increased inflammation and features of atrophic wounds. These results highlight the roles that the AT2R signaling pathway plays in the balance between effective wound healing and chronic wounds.

## 2. Materials and Methods

### 2.1. Mice and Splinted Excisional Wound Model

**Figure 1:**
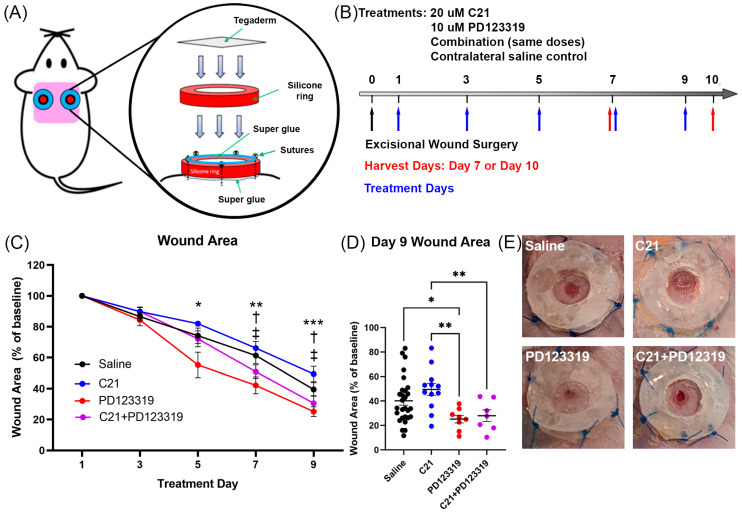
AT2R inhibition during excisional wound healing increases the rate of wound closure. (**A**) Schematic illustration identifying the location and components of the excisional wound–splint apparatus, not to scale. (**B**) Timeline of surgery (black arrow), treatments (blue arrows), and harvests (red arrows). (**C**) Wound area (% baseline ± SEM) over time (treatment day), showing rate of wound closure in wounds treated with saline (black line), 20 µM C21 (blue line), 10 µM PD123319 (red line), and C21 + PD123319 (combination) (purple line). Day 5 * *p* = 0.0142 C21 vs. PD123319; day 7 ** *p* = 0.0038 C21 vs. PD123319, † *p* = 0.0211 saline vs. PD123319, ‡ *p* = 0.0252 C21 vs. combination; and day 9 *** *p* = 0.0007 C21 vs. PD123319, † *p* = 0.0134 saline vs. PD123319, ‡ *p* = 0.0148 C21 vs. combination. Two-way ANOVA with mixed-effects analysis. (**D**) Day 9 wound area (% of baseline) depicted as scatter-dot plots (mean ± SEM), * *p* = 0.0303 saline (*n* = 28) vs. PD123319 (*n* = 8), ** *p* = 0.0026 C21 (*n* = 12) vs. PD123319, ** *p* = 0.0096 C21 vs. combination (*n* = 7), one-way ANOVA with multiple comparisons. (**E**) Representative images of saline (top left), C21 (top right), PD123319 (bottom left), and C21 + PD123319 (bottom right) -treated splinted wounds on day 9.

All animal experiments were performed in accordance with the Canadian Council on Animal Care (CCAC) and approved by the University Committee on Laboratory Animals of Dalhousie University (20-106). Eight-to-ten-week-old male Balb/c (*n* = 8–12/treatment) mice underwent surgery on day 0 as previously described [27]. Briefly, two to three days before surgery, mouse skin was prepared by shaving a section of their upper backs and applying hair removal cream for 30–60 s to prevent early hair regrowth. On day 0, mice were anaesthetized using 2–3% inhaled isoflurane and oxygen. Two full-thickness lesions were created in the dorsal skin cephalad to the scapulae with a 4 mm biopsy punch. Sterilized, 0.5 mm thick silicone rings with an outer diameter of 12 mm and inner diameter of 6 mm (Grace Bio-Labs CultureWell^TM^ silicone isolator sheet material; Millipore-Sigma, Merck, Darmstadt, Germany) were affixed to the skin surrounding the wound using super glue (LePage, Henkel Canada Corporation, Mississauga, ON, Canada) and 5-0 Prolene sutures (Ethicon, Johnson & Johnson, Raritan, NJ, USA). An additional silicone ring was glued over the first ring, providing protection to the sutures and creating a “well” for topical treatments (Figure 1A; all figures copyright 2024 by the authors). Tegaderm film (3M, Saint Paul, MN, USA) was cut to size and placed over both sets of rings and stuck to the surrounding skin.

Topical treatments began on postoperative day 1 and were repeated every second day until sacrifice on days 7 or 10 (Figure 1B). Sterile saline (20 µL) was applied to one wound and 20 µL of one of the following treatments on the other: 20 µM C21 (Axon Medchem, Groningen, The Netherlands); 10 µM PD123319 (APExBIO Technology, Houston, TX, USA); or both C21 and PD123319 (combination treatment). These doses are based on work from our laboratory as well as previously published reports [18,20,23,26,27,28]. Doses were chosen based on related in vitro studies given that treatments were given topically, rather than systemically, as well as pilot in vivo studies in our laboratory. Tegaderm was reapplied after each treatment. High-resolution images were obtained before treatments to monitor wound closure. Wound closure measurements were calculated by calibrating the image scale to a ruler in the image frame using Fiji software (Fiji is Just ImageJ version 1.53q, National Institutes of Health, Bethesda, MD, USA). Wound areas at 3, 5, 7, and 9 days were normalized to the baseline wound area measurement taken of the same wound on day 1 and represented as a percentage. Upon sacrifice, each wound and the surrounding skin were excised and bisected through the wound, with half of the specimen submitted for formalin fixation and the other half stored for RNA extraction.

### 2.2. Scratch Assay

Scratch assays were performed using human dermal fibroblasts (WS1, ATCC, Manassas, VA, USA), human keloid dermal fibroblasts (HKDFs; KEL FIB, ATCC, Manassas, VA, USA), or human epidermal keratinocytes (HEKa, ATCC, Manassas, VA, USA) as previously described [18,21,26]. Cells were seeded into 24-well plates and grown to confluency at 37 °C in a humidified incubator in the appropriate fully supplemented culture medium based on the manufacturers’ instructions. Once confluent, the culture medium was aspirated and replaced with serum-free medium to prevent cell division. After 24 h, the serum-free medium was aspirated and the cells were gently washed with sterile, warmed 1× phosphate-buffered saline (PBS; Gibco, Thermo Fisher Scientific, Waltham, MA, USA). A sterile 200 μL pipette tip was used to create a vertical scratch down the center of the well through the adherent cells. Serum-free media containing one of the following treatments (*n* = 18) was then added to the scratched wells and incubated at 37 °C for 24 h: 5 μM C21; 10 μM C21; 50 μM C21; 10 μM PD123319; or standard serum-free blank medium (untreated control). Based on our previous work and that from other studies, this range of concentrations of C21 and PD123319 do not affect cell viability [18,20,23,26,27,28].

Images of the scratched area were acquired at 0 (immediately after scratch), 24, and 48 h using an EVOSTM XL Core Imaging System (Thermo Fisher Scientific, Waltham, MA, USA). Scratch areas and areas of infiltrating cells were later calculated in Fiji and Microsoft Excel (version 2311, Microsoft, Redmond, Washington, DC, USA) by a blinded observer. Scratch area measurements are represented as a percentage of the initial scratch area at 0 h.

### 2.3. Histology and Multispectral Multiplex Immunofluorescence Staining

For visualization of collagen deposition, a subset of formalin-fixed and paraffin-embedded (FFPE) sections were deparaffinized and underwent Masson’s trichrome staining (Millipore-Sigma, Merck, Darmstadt, Germany) using the overnight post-fixation protocol in Bouin’s solution (Sigma-Aldrich, St. Louis, MI, USA), as per the manufacturer’s instructions. Sections were then dehydrated through a graded series of ethanol incubations and cleared in xylene. Finally, stained sections were mounted and coverslipped with CytosealTM 60 mountant (Thermo Fisher Scientific, Waltham, MA, USA) and allowed to set overnight in a fume hood.

For fluorescent visualization of collagen I (rabbit polyclonal anti-collagen I, Ab21286, 1:500, Abcam, Cambridge, UK), collagen 3 (rabbit polyclonal anti-collagen III, Ab7778, 1:100, Abcam, Cambridge, UK), CD31 (rabbit monoclonal anti-CD31/PECAM-1, clone D8V9E, 77699, 1:200, Cell Signaling, Danvers, MA, USA), CD45 (rabbit monoclonal anti-CD45, clone D3F8Q, 70257, 1:200, Cell Signaling, Danvers, MA, USA), Ly6G (rat anti-Ly6G, clone 1A8, 551459, 1:100, BD Pharmingen, San Diego, CA, USA), and F4/80 (rabbit monoclonal anti-F4-80, clone D2S9R, 70076, 1:100, Cell Signaling, Danvers, MA, USA), we employed multispectral multiplex immunofluorescence staining utilizing the Opal Manual Detection Kit (Akoya Biosciences, Marlborough, MA, USA), optimized for our tissue and targets of interest. FFPE sections were deparaffinized, formalin-fixed overnight, rinsed in dH_2_O, and for each antibody of interest in sequence, underwent antigen retrieval. Antibodies were prepared to their desired working concentrations (see above) in 1× Antibody Diluent/Block, one antibody at a time. Slides were incubated in primary antibody solution for 1 h at room temperature in a humidified chamber, protected from light. Following primary antibody incubation, slides were rinsed in Tris-buffered saline with 0.05% Tween and incubated in 1× Opal Anti-Ms + Rb HRP-conjugate secondary solution. Following additional washes, slides were incubated with a 1:100 solution of the desired Opal reactive fluorophore (Opal 540, 570, 620, or 650) diluted in 1× Plus Amplification Diluent. Slides underwent another series of washes and either began the protocol again for another antibody of interest, or were incubated for 5 min with 4′,6-diamidino-2-phenylindole (DAPI), then rinsed and mounted with Mowiol 4-88 mounting media (Sigma-Aldrich, St. Louis, MI, USA) and left to dry overnight, protected from light. Imaging was always performed on the following day to prevent degradation of the fluorophores.

### 2.4. Collagen and CD31 Imaging Analysis

Brightfield and fluorescent images were acquired using a fluorescence microscope (Olympus BX43, Tokyo, Japan) utilizing Mantra Snap 1.0 software (Akoya Biosciences, Marlborough, MA, USA) and analyzed using inForm Automated Image Analysis software version 2.4.8 (Akoya Biosciences, Marlborough, MA, USA). Fluorescent images underwent background subtraction based on autofluorescence from a skin section subjected to the same staining protocol with the omission of primary antibodies.

Collagen density was calculated from FFPE skin sections stained with Masson’s trichrome and utilizing the built-in color deconvolution tool in Fiji. This process yields red, green, and blue color channels, with the green channel representing the blue collagen stain visible with Masson’s trichrome. A threshold value for positive collagen staining was determined for each section and kept consistent for every image of the same section. Three regions of interest (ROIs) were chosen in a repeatable, unbiased pattern, avoiding section artifact defects and hair follicles in the dermis of each image, and area measurements (limited to threshold) were recorded in Microsoft Excel. Density was calculated by dividing the positive staining area by the area of each ROI to obtain the density for each image. An average collagen density across three images of wounded skin was then normalized by the average collagen density from three images of normal skin.

The ratio of collagen I to III densities was calculated by determining the normalized (wound density/normal density) collagen I and III densities from the same regions in the same fluorescence images. Four normal and four wounded skin images were analyzed per section. In Fiji, three ROIs were selected in a predetermined pattern from the merged images (collagen I, collagen III, and DAPI) to ensure the ROIs were in the dermis. The channels were then split, to yield binary black and white images for each channel. An appropriate threshold value was selected, per antibody, per section, and the same threshold was applied to every image of that channel on the same section. To eliminate differences in staining intensity between sections, all values were normalized between wounded and unwounded skin. The staining area, limited to threshold, was divided by the area of the ROI to obtain the density. The average collagen density from the wounded skin images was then normalized to the average collagen density from the unwounded images. The resultant density for collagen I was divided by that for collagen III, to give the collagen I:III ratio per section (one value per wound). CD31 density was calculated in the same fashion as the collagen I and III densities. Again, the density of CD31 staining in wounded skin was normalized to that in normal skin from the same section.

### 2.5. Mantra Imaging Analysis

For each primary antibody of interest (CD45, Ly6G, and F4/80), a representative batch of 10–15 wound images was selected for training in the inForm software for batch analysis. The treatment (C21, PD123319, or saline control) was unknown to the researcher selecting the images. The following configurations were selected: trainable tissue segmentation (to separate tissue from slide); cell segmentation (to identify cells by nuclei); and phenotyping (to distinguish cells positive or negative for the marker of interest). Tissue segmentation and phenotyping steps involved training the software to recognize the skin tissue from the slide and a positively labelled cell versus one with no positive labeling, respectively. Representative training sets were added to the analysis until >95% accuracy was reached. The project parameters were then saved, and all wound images for each treatment group and antibody run through the same batch analysis.

To determine cellular density, image batch analysis data were analyzed by a blinded observer using the phenoptyrReports (Akoya Biosciences, Marlborough, MA, USA) addition in RStudio (All Other Personal Services, Boston, MA, USA). For each image set (slides stained together), an appropriate threshold for mean staining intensity was selected to distinguish background autofluorescence from a positively labelled cell. As these thresholds varied with staining intensity, density comparisons were compared between each drug treatment and its respective saline control. The number of positively stained cells per image was calculated, yielding a density value based on the area of the region. The cellular densities from each image taken of the same wound were averaged to give an average density per wound. Each treatment group (separated by treatment as well as recovery day) was then normalized to saline, yielding a fold change representing the changes in overall cellular density compared to saline in response to C21 or PD123319 treatments at days 7 and 10.

### 2.6. Quantitative Reverse-Transcription Polymerase Chain Reaction

Skin samples harvested for RNA extraction were stored for 1–7 days in RNAlater^®^ (Sigma-Aldrich, St. Louis, MI, USA), after which the solution was aspirated and the skin samples stored at −80 °C. Frozen skin tissue samples were homogenized in TRIzol (Thermo Fisher Scientific, Waltham, MA, USA) and underwent subsequent chloroform extraction. The aqueous phase containing RNA was purified using the RNeasy Plus Mini Kit (Qiagen, Hilden, Germany) according to the manufacturer’s instructions. RNA concentrations and purity were measured using the Epoch Biotek Microplate Spectrophotometer (Agilent Technologies, Santa Clara, CA, USA).

Isolated RNA then underwent reverse transcription (QuantiTect Reverse Transcription kit, Qiagen, Hilden, Germany) as per the manufacturer’s instructions. Pre-optimized primers (Table 1; copyright 2024 by the authors) for genes of interest (0.25 µM working concentration, per reaction) were prepared in combination with SsoAdvanced Universal SYBR^®^ Green Supermix (5 µL/reaction; Bio-Rad Laboratories, Hercules, CA, USA), sample cDNA (2 µL, 7.5 ng/reaction) and adjusted to a total volume of 10 µL/reaction with RNase-free water. Supermix, primer, and cDNA mixtures were analyzed, in triplicate, using a Bio-Rad CFX96 or CFX384 touch qPCR Real-Time PCR detection system (Bio-Rad Laboratories, Hercules, CA, USA).

All genes of interest were analyzed using the ΔΔCq method, with the Cq values of the gene of interest subtracted by the geometric mean of two reference genes (*Hprt*, hypoxanthine phosphoribosyltransferase 1; *Tbp*, TATA-box binding protein), giving ΔCq, and then normalized expression was calculated as 2^−(ΔCq)^. This value was further normalized to each sample’s respective saline control to yield the fold change.

### 2.7. Statistical Analysis

Data were collected and loaded into Microsoft Excel and analyzed and visualized using GraphPad Prism version 10.0 software (Dotmatics, La Jolla, CA, USA). Data sets were tested for normality using Anderson–Darling, D’Agostino, Shapiro–Wilk, and Kolmogorov–Smirnov tests using the built-in normality detection setting in GraphPad Prism 10.0, and the appropriate parametric or non-parametric tests applied. Comparisons between two means were analyzed using paired or unpaired (as appropriate) t tests (if normality was established), or Mann–Whitney tests. One-way ANOVAs (parametric) or Kruskal–Wallis (nonparametric) tests were used to compare means between more than two groups, and two-way ANOVAs (parametric) or mixed-effects (nonparametric) analyses were used to analyze multiple groups over time. Appropriate post-tests were selected to correct for multiple comparisons. Statistical significance was set as *p* ≤ 0.05.

Where appropriate, control values from saline-treated wounds across the treatment groups were pooled to facilitate analyses. The saline values from each respective treatment pair were statistically compared prior to pooling to ensure no differences between controls and to avoid the potential confounding influence of the diffusion of agents between wounds. No such differences were calculated, so the pooling of saline control values was performed for graphical representation. These data are in Figure 1C,D, Figure 3F, Figure 4B,C and Figure 5B,C.

For analysis of the qPCR data, the reported statistical comparisons are from paired t tests or Wilcoxon tests comparing each pharmacological treatment to its respective saline control only. Normalized gene expression values with respect to saline for each treatment were graphed together. Asterisks represent statistical significance between the indicated treatment group and its saline control. The hashed lines at y = 1 represent the post-normalization saline-treated controls.

## 3. Results

### 3.1. AT2R Signal Inhibition Accelerates Wound Closure in a Mouse Excisional Wound Model

To determine whether AT2R signal modulation during wound healing has an impact on the rate of wound re-epithelialization, wound area was quantified over time and normalized to the initial wound area as measured on treatment day 1. As early as day 5 post-wounding, there was a significant difference in the percentage of wound area relative to baseline between wounds treated with the AT2R agonist, C21, and the AT2R antagonist, PD123319 (Figure 1C). By day 7, PD123319-treated wounds were smaller than saline-treated wounds and those treated with C21 were larger than wounds receiving combination treatment (Figure 1C). By day 10, AT2R blockade with PD123319 resulted in increased wound closure compared to all other treatment groups (Figure 1C–E). No differences were observed in the rate of wound closure between C21- and saline-treated wounds.

### 3.2. AT2R Activation Reduces Human Skin Cell Migration In Vitro

**Figure 2:**
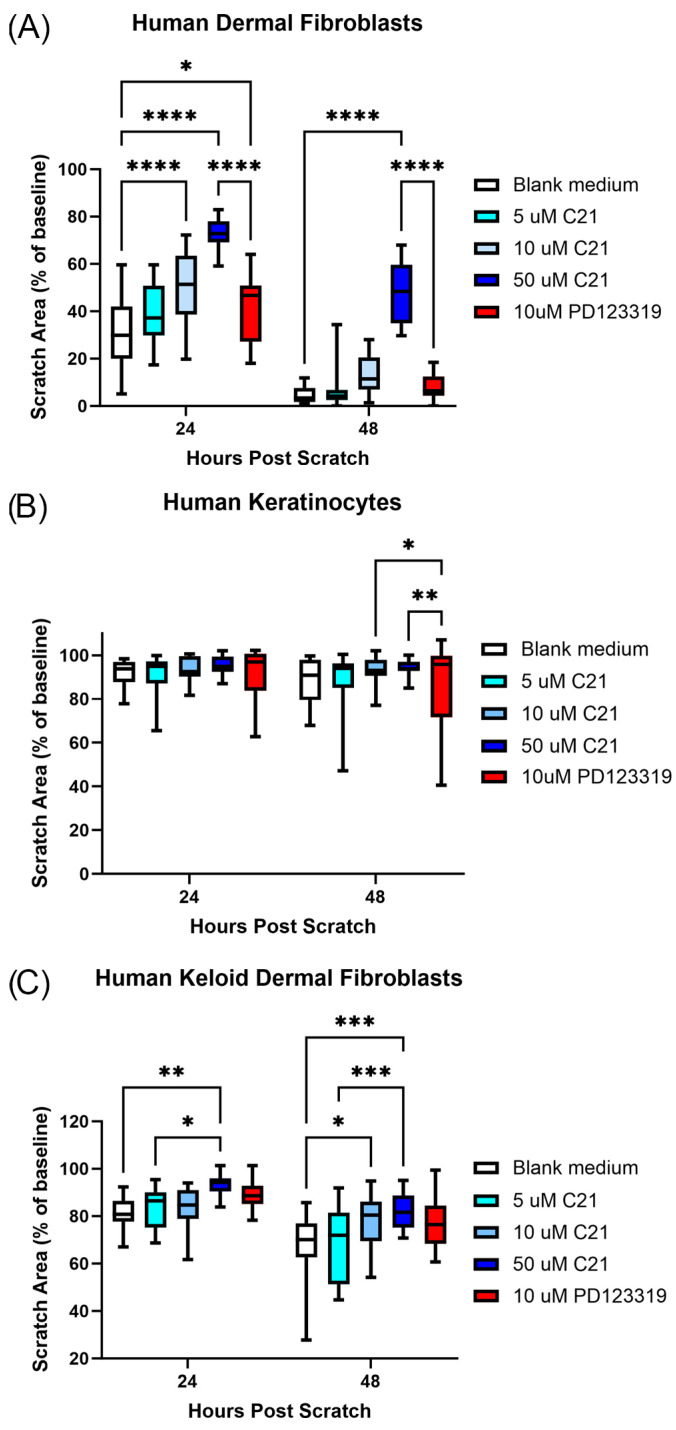
C21 and PD123319 are effective at altering human cell skin cell behavior in vitro. Box and whisker plots (min to max) depicting the scratch area (% of baseline) at 24 and 48 h post scratch (*n* = 18) in (**A**) HDF (WS1), 24 h: **** *p* < 0.0001 blank medium vs. 10 µM C21, **** *p* < 0.0001 blank medium vs. 50 µM C21, * *p* = 0.0375 blank medium vs. 10 µM PD123319, **** *p* < 0.0001 50 µM C21 vs. 10 µM PD123319; 48 h: **** *p* < 0.0001 blank medium vs. 50 µM C21, **** *p* < 0.0001 50 µM C21 vs. 10 µM PD123319, (**B**) HEKa, * *p* = 0.0235 10 µM C21 vs. 10 µM PD123319, ** *p* = 0.0072 50 µM C21 vs. 10 µM PD123319, and (**C**) HKDF, ** *p* = 0.0069 blank medium vs. 50 µM C21 at 24 h, * *p* = 0.0393 5 µM C21 vs. 50 µM at 24 h, * *p* = 0.0348 blank medium vs. 10 µM C21 at 48 h, *** *p* = 0.0005 blank medium vs. 50 µM C21 at 48 h, *** *p* = 0.0009 5 µM C21 vs. 50 µM C21 at 48 h. Mixed-effects analyses with Tukey’s multiple comparisons tests utilized.

To investigate the effects of C21 on human skin cell activity, scratch assays were performed on human dermal fibroblasts (WS1) and keratinocytes (HEKa) (Figure 2A,B, Supplemental Figure S1A,B). In the WS1 cells, both the 10 µM and 50 µM C21 treatments inhibited scratch closure compared to the blank medium control at 24 and 48 h, and 50 µM C21 significantly reduced scratch closure compared to 10 µM PD123319 treatment (Figure 2A). For keratinocytes, PD123319-treated HEKa cells displayed increased migration relative to those treated with 10 µM and 50 µM C21 at 48 h (Figure 2B). However, neither the C21 nor PD123319 treatments differed significantly from untreated controls. Overall, the HEKa cells displayed limited motility. Next, the scratch assays were repeated in human dermal fibroblasts isolated from keloid scars (HKDF), given the well-documented role of AT2R signaling in fibrosis. A similar result was noted in the HKDF cells as in the WS1 cells, with both 10 µM and 50 µM C21 treatments reducing scratch closure compared to blank medium at 24 and 48 h (Figure 2C, Supplemental Figure S1C). An additional dose of 100 µM C21 was attempted on all cell lines but was found to be cytotoxic. Together, these results confirm that human fibroblasts are receptive to AT2R activation via C21, validating future human clinical studies.

### 3.3. Increased AT2R Signaling Increases Early Wound Collagen Density and Reduces Cellular Infiltration

**Figure 3:**
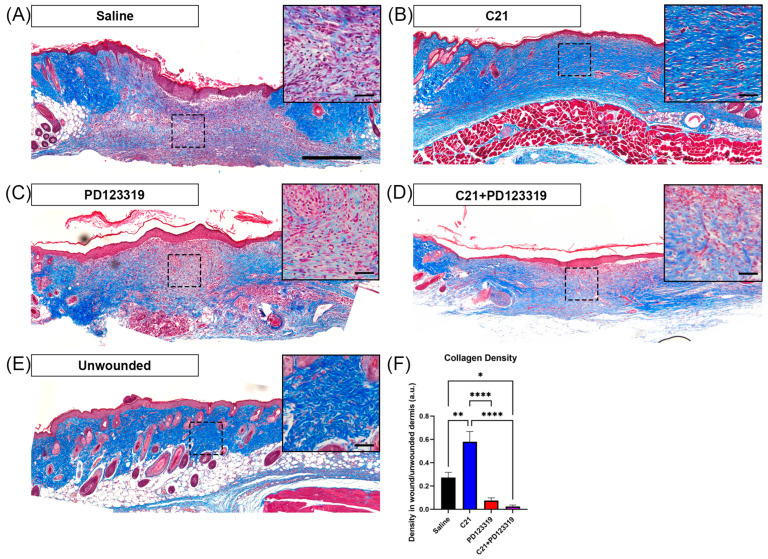
AT2R activation increases wound collagen density. Images acquired at 10× magnification of Masson’s trichrome-stained wounded skin at day 10 post-wounding treated with (**A**) saline (scale = 500 µm), (**B**) 20 µM C21, (**C**) 10 µM PD123319, (**D**) 20 µM C21 + 10 µM PD123319, and (**E**) unwounded skin. Insets (top right, each image) are enlarged sections delineated by the hashed boxes. Inset scale bars = 50 µm. (**F**) Collagen density quantified as wound density normalized to unwounded density (mean + SEM). ** *p* = 0.0011 saline (*n* = 26) vs. C21 (*n* = 11), * *p* = 0.0280 saline vs. C21 + PD123319 (*n* = 8), **** *p* < 0.0001 C21 vs. PD123319 (*n* = 8), **** *p* < 0.0001 C21 vs. C21 + PD123319, one-way ANOVA with Tukey’s multiple comparisons test.

Given the altered fibroblast activity observed in vitro with C21 and PD123319 treatments, we next examined the effects of these compounds on the cellular and structural composition of wounded skin at day 10 in vivo. C21-treated wounds demonstrated collagen densities similar to that of unwounded skin, and greater than those of control or PD123319-treated wounds (Figure 3). Furthermore, wounds treated with the combination of C21 and PD123319 had reduced collagen density compared to both C21 and saline treatments, suggesting that AT2R blockade inhibits early collagen deposition. Interestingly, wounds treated with C21 also demonstrated a striking reduction in cellular infiltration compared to saline, PD123319, and the combination of C21 and PD123319 (Figure 3A,C,D). 

### 3.4. Collagen I:III Ratio Is Increased with AT2R Signaling by Day 10 Post-Wounding

**Figure 4:**
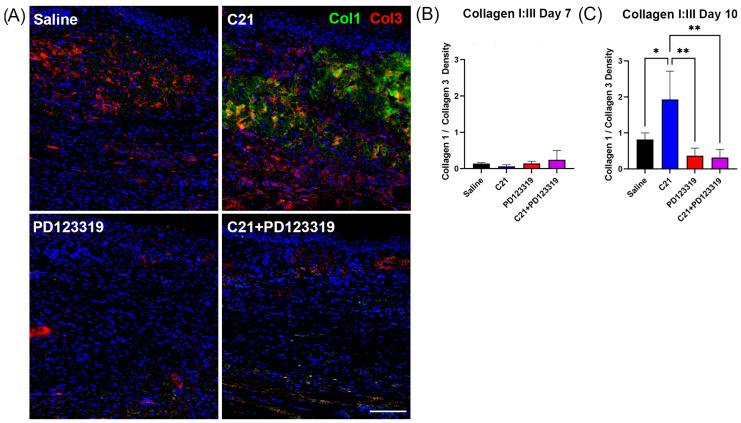
AT2R activation during wound healing improves collagen I:III ratio by day 10. (**A**) Representative immunofluorescence images of wounded skin acquired at 20× stained for collagen 1 (green), collagen 3 (red), and DAPI (blue) and treated with saline (top left), C21 (top right), PD123319 (bottom left), and combination (bottom right). Scale = 100 µm. (**B**) Collagen I:III ratio (mean + SEM) at day 7 post-wounding. C21, PD123319, and combination groups have *n* = 8 replicates, while saline represents *n* = 24, all comparisons are ns, Kruskal–Wallis Test with multiple comparisons. (**C**) Collagen I:III ratio (mean + SEM) at day 10, * *p* = 0.0200 saline (*n* = 23) vs. C21 (*n* = 6), ** *p* = 0.0053 C21 vs. PD123319 (*n* = 8), and ** *p* = 0.0041 C21 vs. combination (*n* = 8), one-way ANOVA with multiple comparisons.

Next, we examined the relative density of different collagen subtypes. It is well established that the ratio of collagen I to collagen III is indicative of skin quality, with wounded skin tending to have lower ratios and unwounded skin or mature wounds yielding higher ones [29,30,31]. We quantified the density of collagens I and III, normalizing each value to normal skin in each section. The ratios of normalized collagen I:III were calculated, and the ratios in unwounded skin per treatment group were confirmed to be equivalent (Supplemental Figure S2B). Similar to our total collagen density results, the collagen I:III ratio was increased in C21-treated wounds relative to saline, demonstrating a ratio more in keeping with unwounded skin at day 10 (Figure 4A,C; Supplemental Figure S2A,B). Additionally, C21-treated wounds had a much higher collagen I:III ratio compared to both PD123319- and combination-treated wounds (Figure 4A,C). To this end, not only were PD123319-treated wounds deficient in total collagen early in the healing process, they also possessed lower ratios of collagen I:III than unwounded or C21-treated skin, akin to immature wounds. Further, when the normalized densities of collagen I and III were compared, PD123319 and combination-treated wounds still displayed unfavorably high levels of collagen III relative to collagen I at day 10, while C21-treated wounds did not (Supplemental Figure S2C,D).

Given the rapid rate of wound closure observed with PD123319 treatment, we chose to examine an earlier time point (7 days post-wounding) to investigate earlier cellular and extracellular changes. No significant differences in the collagen I:III ratio were observed between any treatment groups at day 7 (Figure 4B), suggesting that the advanced remodeling effects observed in C21-treated wounds were likely to occur between days 7 and 10 post-wounding in mice.

### 3.5. AT2R Activation Increases Wound Vascular Density

**Figure 5:**
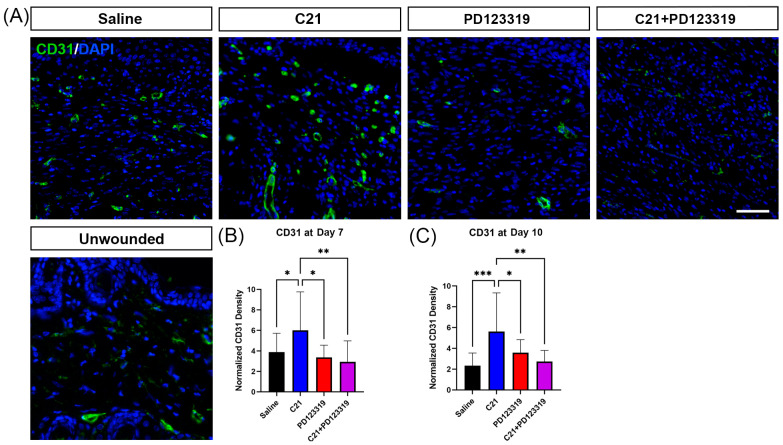
AT2R activation during wound healing increases CD31 staining density. (**A**) Immunofluorescence images acquired at 20× of day 10 wounded skin stained for CD31 (green) and DAPI (blue), scale = 50 µm. (**B**) CD31 density normalized to unwounded skin (mean + SD) at day 7 post-wounding, * *p* = 0.0230 saline (*n* = 24) vs. C21 (*n* = 8), * *p* = 0.0217 C21 vs. PD123319 (*n* = 8), ** *p* = 0.0080 C21 vs. combination (*n* = 8), one-way ANOVA with multiple comparisons. (**C**) CD31 density normalized to unwounded skin (mean + SD) at day 10 post-wounding, *** *p* = 0.001 saline (*n* = 22) vs. C21 (*n* = 8), * *p* = 0.0441 C21 vs. PD123319 (*n* = 7), ** *p* = 0.0038 C21 vs. combination (*n* = 8), one-way ANOVA with multiple comparisons.

Angiogenesis and vasculogenesis are hallmarks of early wound healing, promoting cell migration, collagen crosslinking, and wound maturation [32]. Therefore, we examined the effects of AT2R signal modulation on wound vascular density. As early as day 7 post-wounding, C21-treated wounds had higher staining densities of the blood vessel endothelial cell marker CD31 relative to the saline, PD123319, and combination treatments (Figure 5B). This pattern persisted on day 10 (Figure 5A,C), indicating that AT2R activation promotes angiogenesis by day 7 post-wounding, an important feature of healthy wound healing.

### 3.6. AT2R Signal Modulation Alters Wound Remodeling and Immune Cell Gene Transcription

**Figure 6:**
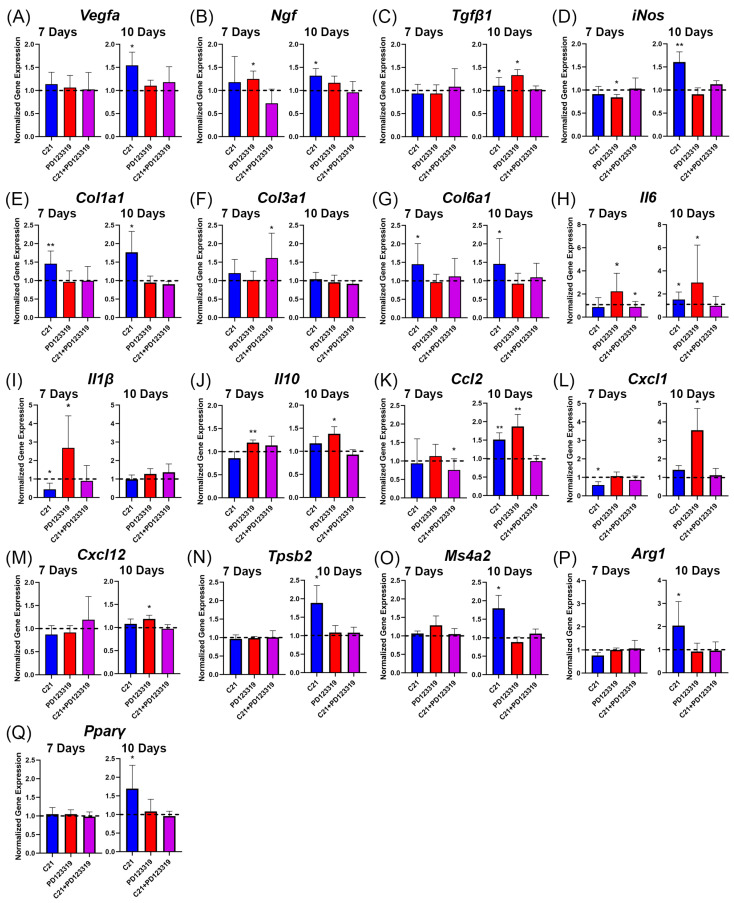
Fold change of gene expression (mean + SEM) relative to saline (delineated by the hashed lines) at days 7 and 10 post-wounding. Paired *t*-tests (or Wilcoxon) between treatment and respective saline control were utilized. Asterisks indicate pairwise comparisons to saline (represented by the hashed lines at y=1). (**A**) Vegfa (*n* = 8), * *p* = 0.0092 C21 vs. saline at day 10. (**B**) Ngf (day 7 C21 and PD123319 *n* = 8, combination *n* = 7; day 10 C21 *n* = 11, PD123319 *n* = 7, combination *n* = 8), * *p* = 0.0013 PD123319 vs. saline at day 7 and * *p* = 0.0399 C21 vs. saline at day 10. (**C**) Tgfβ1 (day 7 C21 and PD123319 *n* = 8, combination *n* = 7; day 10 C21 and combination *n* = 8, PD123319 *n* = 7), * *p* = 0.0429 C21 vs. saline and * *p* = 0.0391 PD123319 vs. saline at day 10. (**D**) iNos (day 7 C21 and PD123319 *n* = 8, combination *n* = 7; day 10 all *n* = 8), * *p* = 0.0264 PD123319 vs. saline at day 7 and ** *p* = 0.0029 C21 vs. saline at day 10. (**E**) Col1a1 (day 7 all *n* = 8; day 10 C21 *n* = 12, PD123319 and combination *n* = 8), ** *p* = 0.0042 C21 vs. saline at day 7 and * *p* = 0.0441 C21 vs. saline at day 10. (**F**) Col3a1 (day 7 C21 *n* = 7, PD123319 and combination *n* = 8; day 10 C21 *n* = 9, PD123319 *n* = 7, combination *n* = 8), * *p* = 0.0123 C21 + PD123319 vs. saline at day 7. (**G**) Col6a1 (day 7 all *n* = 8; day 10 C21 *n* = 9, PD123319 *n* = 7, combination *n* = 8), * *p* = 0.0323 C21 vs. saline at day 7 and * *p* = 0.0391 C21 vs. saline at day 10. (**H**) Il6 (day 7 C21 and PD123319 *n* = 8, combination *n* = 7; day 10 C21 *n* = 9, PD123319 and combination *n* = 8), * *p* = 0.0324 PD123319 vs. saline and * *p* = 0.0419 C21 vs. PD123319 at day 7, * *p* = 0.0467 C21 vs. saline and * *p* = 0.0391 PD123319 vs. saline at day 10. (**I**) Il1β (day 7 all *n* = 8; day 10 C21 *n* = 9, PD123319 *n* = 7, combination *n* = 8), * *p* = 0.0066 C21 vs. saline and * *p* = 0.0220 PD123319 vs. saline at day 7. (**J**) Il10 (all *n* = 8), ** *p* = 0.0039 PD123319 vs. saline at day 7 and * *p* = 0.0307 PD123319 vs. saline at day 10. (**K**) Ccl2 (all *n* = 8), * *p* = 0.0198 C21 + PD123319 vs. saline at day 7, and ** *p* = 0.0063 C21 vs. saline and ** *p* = 0.0148 PD123319 vs. saline at day 10. (**L**) Cxcl1 (day 7 C21 *n* = 8, PD123319 and combination *n* = 7; day 10 C21 *n* = 7, PD123319 and combination *n* = 8), * *p* = 0.0129 C21 vs. saline at day 7 and * *p* = 0.0269 PD123319 vs. saline at day 10. (**M**) Cxcl12 (all *n* = 8), * *p* = 0.0281 PD123319 vs. saline at day 10. (**N**) Tpsb2 (day 7 C21 and PD123319 *n* = 8, combination *n* = 7; day 10 C21 *n* = 9, PD123319 and combination *n* = 8), * *p* = 0.0469 C21 vs. saline at day 10. (**O**) Ms4a2 (day 7 all *n* = 8; day 10 C21 *n* = 10, PD123319 and combination *n* = 8), * *p* = 0.0195 C21 vs. saline at day 10. (**P**) Arg1 (day 7 all *n* = 8; day 10 C21 *n* = 6, PD123319 *n* = 8, combination *n* = 7), * *p* = 0.0345 C21 vs. saline at day 10. (**Q**) Pparγ (day 7 all *n* = 8; day 10 C21 and PD123319 *n* = 8, combination *n* = 7), * *p* = 0.0114 C21 vs. saline at day 10.

To assess the molecular pathways related to AT2R signaling, we employed quantitative reverse-transcription qPCR (RT-qPCR) to determine the expression of a variety of genes involved in wound healing, remodeling, and immune cell signaling. In keeping with the CD31 staining results, transcription of vascular endothelial growth factor (*Vegfa*) was increased with C21 treatment relative to saline at day 10 but not at day 7 (Figure 6A). To assess wound reinnervation and returned sensation, we measured nerve growth factor (*Ngf*) gene expression. The NGF protein functions to promote axon regeneration into the wound area and is associated with increased skin functional recovery as well as promoting fibroblast migration and proliferation [33]. *Ngf* was elevated relative to saline with PD123319 treatment on day 7 and with C21 treatment at day 10 (Figure 6B). Transforming growth factor β subunit 1 (*Tgfβ1*) transcript levels, which are known to increase during early wound healing to regulate inflammation and stimulate collagen accumulation [34], were unaffected by either compound relative to saline at day 7, with modest increases at day 10 observed with C21 and PD123319 treatments (Figure 6C). C21 also increased the transcription of inducible nitric oxide synthase (*iNos*) at day 10 (Figure 6D). INOS stimulates the production of nitric oxide (NO), which in turn has been shown to regulate pro-fibrotic TGF-β signaling [35,36], but is also essential for healthy collagen production during wound healing [37]. Additionally, *iNos* was downregulated by PD123319 treatment at day 7 (Figure 6D).

To determine whether our observed changes in collagen composition with AT2R signal modulation agree with the gene expression, we analyzed the wounded skin samples for the genes collagen type I α 1 (*ColIa1*), collagen type 3 α 1 (*Col3a1*), and collagen type 6 α 1 (*Col6a1*), the latter of which is associated with collagen organization and remodeling. In keeping with our immunofluorescence findings for the collagen I:III ratio, C21-treated wounds demonstrated upregulated transcript levels of *Col1a1* relative to saline at day 7 and day 10 (Figure 6E). There was no corresponding increase in *Col3a1* expression with C21 treatment at either time point. Interestingly, there was increased expression of *Col3a1* with the combination treatment of C21 and PD123319 at day 7 (Figure 6F). C21 treatment also increased transcription of the remodeling gene *Col6a1* at both time points, indicating that at day 7 post-wounding, C21-treated wounds expressed markers of wound remodeling earlier than saline-treated wounds (Figure 6G).

Next, we analyzed the transcription levels of genes encoding the pro-inflammatory interleukins 6 (IL6) and 1β (IL1β) and the anti-inflammatory interleukin 10 (IL10). *Il6* gene expression was upregulated in PD123319-treated wounds both at day 7 and day 10 (Figure 6H). In keeping with its role in the early inflammatory phase, differences in *Il1β* were only observed at day 7 post-wounding, with C21 leading to downregulation and PD123319 to upregulation (Figure 6I). Surprisingly, the transcription levels of anti-inflammatory *Il10* were also increased in PD123319-treated wounds both at day 7 and day 10 (Figure 6J).

To explore the nature of the observed cellular infiltration in the wounds treated with PD123319 or C21, we analyzed the expression of various immune cell markers and chemoattractant-encoding genes. The gene C-C motif chemokine ligand 2 (*Ccl2*) encodes monocyte chemoattractant protein (MCP) [38,39,40] and was found to be upregulated by both C21 and PD123319 at day 10, with the upregulation noted to be greater with the latter (Figure 6K). *Cxcl1* encodes C-X-C motif chemokine ligand 1 (CXCL1), which has well-documented roles in inflammation, angiogenesis, and wound healing, including serving as a chemoattractant for neutrophils [40,41]. It was downregulated in C21-treated wounds at day 7 and significantly upregulated at day 10 in PD123319-treated wounds (Figure 6L). C-X-C motif chemokine ligand 12 (*Cxcl12*) encodes stromal derived factor (SDF), a lymphocyte chemoattractant [41,42,43], and was found also to be upregulated in PD123319-treated wounds at day 10 (Figure 6M).

Mast cells are known to be key regulators of wound healing, exhibiting various roles depending on their release of different factors [7,44,45]. Mast cells are responsible for recruiting neutrophils and monocytes during early wound healing and also participate in angiogenesis and ECM synthesis [7]. We found that C21 increased the gene expression of the mast cell markers tryptase β 2 (*Tpsb2*) and membrane-spanning 4-domains, subfamily A, member 2 (*Ms4a2*) at day 10 post-wounding (Figure 6N,O). Finally, as macrophages represent a heterogeneous cell population, we examined expression of two genes associated with anti-inflammatory macrophage activity, arginase 1 (*Arg1*) and peroxisome proliferator-activated receptor γ (*Pparγ*) [46], and found increased expression of both genes with C21 treatment at day 10 (Figure 6P,Q).

### 3.7. Increased AT2R Signaling during Wound Healing Promotes an Anti-Inflammatory Immune Cell Microenvironment

**Figure 7:**
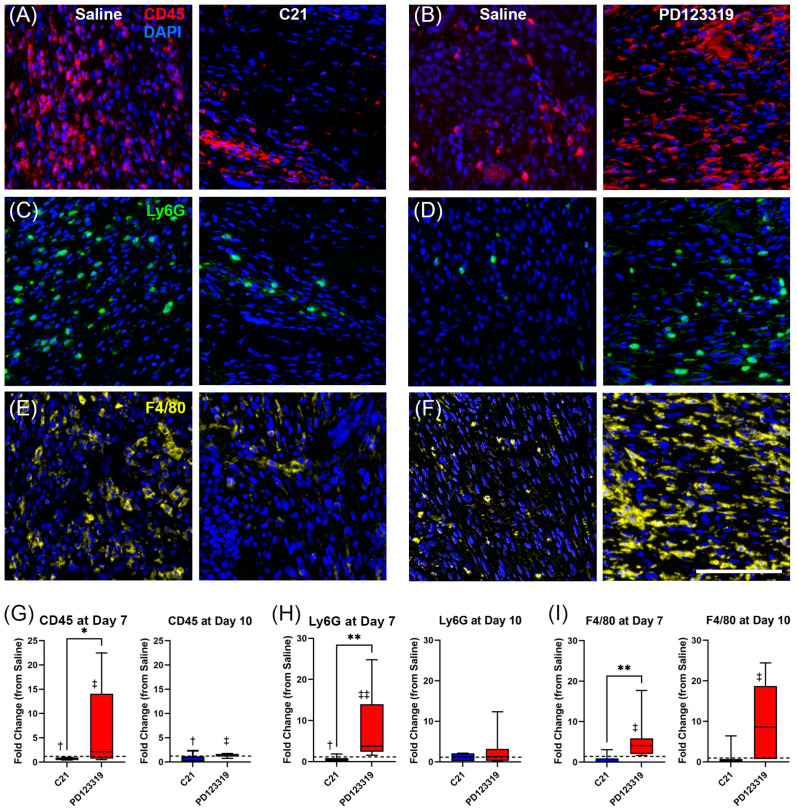
AT2R signaling alters the density of immune cell types in healing wounds. Representative 20X fluorescence images of CD45-positive cells (red) and DAPI (blue) labelling all nuclei in (**A**) saline- (left)/C21- (right) and (**B**) saline- (left)/PD123319- (right) treated wound pairs; Ly6G-positive cells (green) and DAPI in (**C**) saline- (left)/C21- (right) and (**D**) saline- (left)/PD123319- (right) treated wounds; and F4/80-positive cells (yellow) and DAPI in (**E**) saline- (left)/C21- (right) and (**F**) saline- (left)/PD123319- (right) treated wounds. All images acquired at day 7. (**G**) Density of CD45-positive cells normalized to respective saline controls, represented as fold change (box and whisker) in C21- (*n* = 8; blue) and PD123319- (*n* = 7; red) treated wounds vs. saline at days 7 and 10 post-wounding (* *p* = 0.0379 normalized C21 vs. PD123319 at day 7; † C21 vs. saline at day 7 *p* = 0.0469, and day 10 *p* = 0.0348; ‡ PD123319 vs. saline at day 7 *p* = 0.0287, and day 10 *p* = 0.0280). (**H**) Density of Ly6G-positive cells normalized to saline (box and whisker) in C21- (*n* = 7; blue) and PD123319- (*n* = 7; red) treated wounds at days 7 and 10 (** *p* = 0.0012 normalized C21 vs. PD123319; † *p* = 0.0499 C21 vs. saline day 7; ‡‡ *p* = 0.0026 PD123319 vs. saline day 7). (**I**) Density of F4/80-positive cells normalized to saline (box and whisker) of C21- (*n* = 6; blue) and PD123319- (*n* = 7; red) treated wounds at days 7 and 10 post-wounding (** *p* = 0.0047 normalized C21 vs. PD123319; ‡ *p* = 0.0297 and 0.0428 PD123319 vs. saline at days 7 and 10, respectively). Hashed lines at y=1 indicate saline-treated controls. Paired t and Mann–Whitney tests used between treatments and saline controls. Between-treatment comparison of normalized values analyzed with unpaired t and Mann–Whitney tests. Scale bar = 100 µm.

The Masson’s trichrome staining and gene expression analyses support the possibility of changes in immune cell numbers and subtypes within wounds treated with C21 and PD123319. Quantitative multiplex immunofluorescence analysis was therefore employed to examine specific cell types within the wound. We sought to determine whether AT2R signal modulation during wound healing leads to different densities of immune cell types, as our qPCR results suggest. We stained sections of wounded skin with the immune cell markers, CD45 (leukocyte common antigen, all hematopoietic cells, except erythrocytes), Ly6G (lymphocyte antigen 6 family member G, neutrophils), and F4/80 (EGF-like module containing mucin-like hormone receptor-like 1, macrophages) (Figure 7A–F). Considering that no significant differences were observed between PD123319- and combination-treated groups for our earlier measures, we proceeded to quantify the immune cell subpopulation densities in wounds treated with C21 and PD123319 only, with their respective saline controls.

We found that C21 treatment significantly reduced the density of CD45+ hematopoietic cells both at day 7 and day 10 relative to saline (Figure 7A,G). In contrast, treatment with PD123319 increased the density of CD45+ cells at both time points (Figure 7B,G). In terms of individual leukocyte populations, AT2R activation with C21 decreased the density of Ly6G+ neutrophils in the wounded skin at day 7 (Figure 7C,H), while PD123319-treated wounds had an increased density of Ly6G+ cells relative to saline (Figure 7D,H). In keeping with the appearance and clearance of neutrophils in the early inflammatory phase of healing [4,41], the Ly6G results were not significant at day 10. For macrophages, C21 treatment did not reduce F4/80+ cell density at day 7 or day 10 (Figure 7E,I). In contrast, at both days 7 and 10 post-wounding, treatment with PD123319 yielded a much greater F4/80 staining density relative to saline (Figure 7F,I). Interestingly, the fold changes in immune cell density relative to saline between C21- and PD123319-treated wounds were significantly different for all immune cell markers tested, with PD123319-treated wounds consistently displaying greater fold changes at day 7 (Figure 7B,D,F–I). Overall, by day 10 post-wounding, C21-treated wounds had either reduced (CD45) or unchanged (Ly6G and F4/80) densities of immune cells relative to saline, while PD123319-treated wounds had significantly increased densities of all immune cell markers relative to saline (Figure 7G–I).

## 4. Discussion

Our results support a role for the AT2R signaling pathway in re-epithelialization, as wound closure was increased when AT2R signaling was inhibited with PD123319 (Figure 1C–E). Conversely, AT2R activation via C21 treatment improved collagen composition, vascular density, and dramatically reduced inflammation (Figure 3, Figure 4, Figure 5, Figure 6 and Figure 7). Indeed, C21 appeared to accelerate the rate of wound healing relative to other treatments, with cellular and structural features closer to those observed in unwounded skin. In contrast, while wounds treated with PD123319 re-epithelialized more rapidly, this was at the expense of wound quality, with evidence of increased inflammation and poor extracellular matrix structure. Importantly, the effects of C21 appeared to be AT2R specific, as combination-treated wounds did not differ from those treated with the inhibitor alone. Our results are in keeping with previous research where wound healing was examined in AT2R null mice [47]. Similar to our results with PD123319 treatment, that study found that mice lacking AT2R had accelerated wound closure, but their wounds were structurally more fragile on biomechanical tension and elongation assessments [47].

In vitro, C21 treatment had a more robust effect on limiting migration in the two human fibroblast cell lines relative to the keratinocytes. We found robust dose-response effects for C21 on both human fibroblast cell lines used, particularly the WS1 cells (Figure 2A,C), supporting previous findings [18]. C21 was less effective at inhibiting the activity of the HKDF cells than the WS1 cells, perhaps due to the former’s increased fibrotic activity. Additionally, we found that that C21 did not alter keratinocyte migration compared to blank medium controls (Figure 2B), which agrees with our finding that C21 also did not alter the rate of re-epithelialization in vivo (Figure 1C,D). Interestingly, keratinocyte migration in vitro has been reported to be slowed both by AT1R and AT2R inhibition, indicating that AngII signaling in keratinocytes may be independent of a pro- or anti-fibrotic phenotype [26]. While we did not observe the same, here, numerous factors in addition to keratinocyte migration contribute to re-epithelialization in vivo, including proliferation of epithelial stem cells and fibroblasts and ECM deposition [1,4,48]. Additionally, keratinocytes become activated in vivo through exposure to mechanical stimuli, cytokine signaling, oxidative stress, and growth factors, none of which were present in vitro [1,4,48]. These mechanisms likely contributed to the increased re-epithelialization rate that we observed with PD123319 treatment in vivo (Figure 1C,D). Further analysis of alternative keratinocyte cell lines and doses of PD123319 are warranted to conclusively determine the influence of this compound on keratinocyte motility. However, our results that PD123319 treatment did not inhibit fibroblast infiltration into the scratch area is supported by previous work in primary HKDF cells that 100 µM, a much higher dose than the present study, did not significantly alter migration (Figure 2A,C) [49]. Furthermore, topical treatment with the AT2R inhibitor in the present study yielded the same rapid re-epithelialization reported by Faghih and colleagues (2015) in AT2R null mice [47]. Together, our in vitro results support that C21 is effective at altering fibroblast but not keratinocyte activity, while our in vivo results support that pharmaceutical AT2R blockade accelerates re-epithelialization. Importantly, we have shown that C21 and PD123319 are safe and effective at altering human skin cell behavior, providing essential proof-of-concept evidence for future clinical applications.

Our results also revealed increased collagen content closer to that of unwounded skin with AT2R activation via C21 relative to either saline and PD123319 treatment in vivo (Figure 3 and Figure 4). C21 treatment also significantly improved the collagen I:III ratio even in comparison to saline treated wounds, indicating that increasing AT2R signaling may speed up the structural remodeling phase of wound healing. These findings of improved collagen density and the ratio of collagen I:III were only observed at day 10 post-wounding (Figure 3, Figure 4 and Figure S2). However, we did observe increased angiogenesis as early as day 7 (Figure 5), which could explain why the collagen I:III ratios only increased by day 10. Increased perfusion has several benefits in wound healing, including improved ATP generation, enhanced neutrophil anti-bacterial function, improved fibroblast migration and function, and promotion of collagen crosslinking and deposition [32,40,50]. Wound hypoxia is also causative of chronic wounds [32,51]. Furthermore, topical oxygen and hyperbaric oxygen therapies have long been utilized in clinical practice to improve wound healing [40,51,52]. Therefore, our results of increased vascular density in advance of increased collagen density with C21 treatment support the concept that increased wound oxygen facilitates fibroblast function and collagen cross-linking (Figure 5A–C). Additionally, C21 treatment resulted in increased transcript levels of two mast cell-associated genes, *Tpsb2* and *Ms4a2* (Figure 6N,O), and mast cells are known to contribute to angiogenesis and new capillary bed formation [4,7]. Together, this provides further evidence that augmenting AT2R signaling during wound healing accelerates wound maturation. Interestingly, we were only able to detect increased *Vegfa* gene transcript expression at day 10 (Figure 6A), an observation that could be attributed to a positive feed-forward mechanism resulting in increasing angiogenesis over recovery time [40]. The same pattern of *Vegfa* gene transcription was also observed in the transcript levels of *Arg1* and *Pparγ* at day 10, both of which are associated with a pro-reparative (or M2) macrophage phenotype [53,54,55]. These findings agree with the well-established role of canonical M2 (healing-associated) macrophages in promoting angiogenesis [56,57,58].

Increased *Ngf* gene expression with PD123319 treatment at day 7 and with C21 at day 10 post-wounding suggests that different mechanisms promote *Ngf* expression at different time points during wound healing (Figure 6B). Additionally, aberrant NGF protein activity, especially when associated with inflammation, can be indicative of neuropathic pain [59,60]. Indeed, increased *Ngf* expression in conjunction with increased *Il1β* and *Il6* expression are common reported features of neuropathic pain [59,60]. Here, wounds treated with PD123319 had a similar gene expression profile at day 7 post-wounding (Figure 6B,H,I), suggesting that blockade of AT2R signaling during wound healing results in a pathological wound environment. Increased *Ngf* with C21 treatment at day 10 was also associated with a modest increase in *Il6* but not *Il1β* (Figure 6B,H,I). Neurite regeneration during wound healing is slow, and is more active in the late proliferative and remodeling phases [33,61]. As cellular and inflammatory debris clears, these delicate structures are more able to successfully reach their targets [33]. We speculate that increased *Ngf* at this later time point could indicate increased neurite regeneration rather than neuropathic pain in the C21-treated wounds. Indeed, AT2R activation has long been implicated in the regulation of pain by suppressing inflammation and reactive oxygen species, while promoting tissue repair and anti-nociceptive signaling [62,63,64,65,66,67].

The gene expression of *Tgfβ1* was also robustly increased at day 10 in wounds treated with the AT2R antagonist, indicative of a pro-fibrotic phenotype, with long-term *Tgfβ1* transcription in wound repair being associated with progressive fibrosis and scarring (Figure 6C) [34]. Mechanistically, previous reports have tied elevated TGF-β1 levels to chronic fibrotic conditions by contributing to epithelial senescence, endoplasmic reticulum stress, and mesenchymal transition [34]. We did not observe a similar reduction in *Tgfβ1* transcription with AT2R activation in vivo as has been previously described in vitro [18] and instead saw a modest increase (Figure 6C). However, while TGF-β1 has an established role in promoting fibrosis and collagen accumulation, it has similarly well-documented roles in the anti-inflammatory response and resolving inflammation [68,69]. Indeed, excision of *Tgfβ1* is lethal in mice, resultant of overwhelming systemic inflammation [70]. Therefore, the exact mechanism whereby C21 alters TGF-β to reduce fibrosis bears further investigation, as it is clear that intricate regulation of this pathway can tip the balance towards, or away from, fibrosis.

A study examining the effect of AT2R stimulation in a rat model of hypertension found that C21 reduced renal inflammation, increased the production of NO and cyclic GMP, and decreased the expression of TGF-β [71]. This agrees with our finding of robustly increased transcription level of *iNos* with C21 treatment at day 10 post-wounding (Figure 6D). NO has previously been shown to inhibit TGF-β by reducing secreted modular calcium-binding protein 1 (SMOC-1) in kidney mesangial cells [35]. Additionally, TGF-β-induced synthesis of excessive ECM proteins in osteoarthritis was reduced by NO [72]. However, in contrast, NO has also been speculated to contribute to the aberrant signaling that contributes to fibrosis in keloid scars and is a canonical marker of pro-inflammatory macrophages [56,58,73]. Inhibitors of nitric oxide synthase have also been shown to downregulate the expression of TGF-β and phospho-Smad2/3, a downstream effector of TGF-β, in fibroblast cultures [36]. These results suggest that TGF-β signaling may be downstream of NO signaling [36]. Given that numerous studies report NO activity in conjunction with TGF-β signaling and that we observed increased *iNos* with C21 (Figure 6D) [71], C21 anti-fibrotic activity may be NO dependent. Our results support the concept that C21 induces NO synthase, which in turn increases NO, which may inhibit the fibrotic activity of TGF-β in the setting of cutaneous wound healing.

Our findings of increased inflammatory cells and mediators in PD123319-treated wounds with the opposite observed in C21-treated wounds were striking. We observed increased pro-inflammatory *Il6* gene expression at both days 7 and 10, and *Il1β* at day 7 post-wounding in PD123319-treated wounds (Figure 6H). Additionally, the increase in Ly6G+ neutrophils with PD123319 treatment relative to saline at day 7 (Figure 7D,H) was particularly intriguing, given the well-documented contribution of neutrophils to the chronic wound environment [4,6]. Given the rapid clearance of neutrophils from the wound site via apoptosis by 4 or 5 days post-wounding, the lack of a significant difference in neutrophil numbers by day 10 was not surprising [4,6]. However, importantly, C21 treatment reduced neutrophil density and *Cxcl1* expression relative to saline at day 7 (Figure 6L and Figure 7C,H).

In terms of macrophages, we observed increased F4/80 staining density with PD123319 treatment (relative to saline) at both time points, indicative either of increased numbers of wound macrophages or differential macrophage activation in response to AT2R inhibition (Figure 7F,I). The finding of increased F4/80 staining density with PD123319 also correlates with the increased gene expression of *Il6* and *Tgfβ1* with the same treatment (Figure 6C,H), both of which are known to be produced by macrophages, as well as *Ccl2*, which encodes MCP (Figure 6K) [8,9]. Interestingly, the increase in these macrophage-associated pro-inflammatory mediators with PD123319 was not observed in terms of *iNos* gene expression, which instead was increased with C21 treatment at day 10 (Figure 6D). This suggests an alternative mechanism for stimulating the NO pathway. Indeed, AT2R blockade has previously been shown to reduce *iNos*, despite promoting fibrosis, agreeing with our results [74]. Blocking NO synthesis has also been shown to impair wound healing due to impaired collagen synthesis and reduced fibroblast proliferation [37]. Further, M1 macrophages are not the only source of *iNos*, with fibroblasts and keratinocytes known to produce it as well [75]. Our results also support that C21 promotes collagen synthesis and angiogenesis at day 10, both of which are known to be associated with increased *iNos* [37,75]. These results suggest that AT2R signal modulation during cutaneous wound healing impacts the localization and activity of immune cell subpopulations. Indeed, overall CD45+ immune cell numbers were increased in PD123319-treated wounds and reduced in wounds treated with C21 at both time points (Figure 7A,B,G).

Together, these observations show that inhibition of AT2R during wound healing results in rapid but poor healing with features of chronic, atrophic wounds, while AT2R activation with C21 results in markedly reduced inflammation and a rapid restoration of collagen density approximating unwounded skin.

## 5. Conclusions

The current study highlights the role of AT2R signaling in promoting wound healing. When compared to control or AT2R inhibition, AT2R activation with C21 resulted in wounds with decreased inflammatory cell infiltration and pro-inflammatory gene transcription, near-normal collagen density and collagen I:III ratio, increased vascular density, and increased expression of genes promoting tissue remodeling. Strikingly, the reduced cellular infiltrate and collagen compositions in C21-treated wounds approximated those of unwounded skin. These robust changes were observed between days 7 and 10 of healing, correlating with the transition from the proliferative to the remodeling phases. Furthermore, high concentrations of C21 were safe and effective at influencing human skin cell activity in vitro, a finding which could lay the groundwork for a future phase 1 clinical trial examining C21 in human wound healing. Together, these preclinical findings provide a proof-of-principle for the use of topical C21 as a potential therapeutic agent to improve wound healing.

## Figures and Tables

**Table 1:** List of primers used.

Gene Name	Supplier	Location
*Arg1*	Bio-Rad Laboratories	Hercules, CA, USA
*Ccl2*	Bio-Rad Laboratories	Hercules, CA, USA
*Col1a1*	Invitrogen (Thermo Fisher)	Waltham, MA, USA
*Col3a1*	Invitrogen (Thermo Fisher)	Waltham, MA, USA
*Col6a1*	Bio-Rad Laboratories	Hercules, CA, USA
*Cxcl1*	Qiagen	Hilden, Germany
*Cxcl12*	Qiagen	Hilden, Germany
*Il10*	Invitrogen (Thermo Fisher)	Waltham, MA, USA
*Il1β*	Invitrogen (Thermo Fisher)	Waltham, MA, USA
*Il6*	Bio-Rad Laboratories	Hercules, CA, USA
*iNos*	Invitrogen (Thermo Fisher)	Waltham, MA, USA
*Ms4a2*	Qiagen	Hilden, Germany
*Ngf*	Bio-Rad Laboratories	Hercules, CA, USA
*Pparγ*	Bio-Rad Laboratories	Hercules, CA, USA
*Tgfβ1*	Qiagen	Hilden, Germany
*Tpsb2*	Qiagen	Hilden, Germany
*Vegfa*	Invitrogen (Thermo Fisher)	Waltham, MA, USA
*Hprt*	Integrated DNA Technologies	Coralville, IA, USA
*Tbp*	Integrated DNA Technologies	Coralville, IA, USA

## Data Availability

Pertinent data generated or analyzed during the current study are included in this published article. Any additional raw datasets are available from the corresponding author upon request.

## Supplementary Materials

The following supporting information can be downloaded at: https://www.mdpi.com/article/10.3390/biomedicines12061238/s1, Figure S1: Scratch assays in human skin cell culture. Representative brightfield images acquired at 10× of scratched areas at 24 and 48 hours in (**A**) human dermal fibroblasts (HDF), (**C**) human keratinocytes (HEKa) (**E**) human keloid dermal fibroblasts (HKDF) treated with 5 µM C21, 50 µM C21, 10 µM PD123319, or blank medium. Scale = 250 µm; Figure S2: Collagen I and III densities in wounded and unwounded skin. (**A**) Fluorescence image of unwounded skin at day 10 acquired at 20x and stained for collagen I (Col 1; green), collagen III (Col 3; red), and DAPI (blue). Scale bar = 100 µm. (**B**) Ratio of collagen I:collagen III for unwounded skin from each treatment group at day 10 (mean ± SEM), all comparisons ns. Normalized densities (mean ± SEM) of collagen I and III for wounded skin in each treatment group at (**C**) day 7 (* *p* < 0.000001 saline Col I vs. Col III, * *p* = 0.00016 C21 Col I vs. Col III, * *p* = 0.00031 PD123319 Col I vs. Col III and * *p* = 0.0148 combination Col I vs. Col III) and D) day 10 post-wounding (* *p* = 0.0104 PD123319 Col I vs Col III and * *p* = 0.0047 combination Col I vs Col III). Multiple Mann Whitney tests. Showing lesser densities of mature collagen type I in PD123319 and combination treated groups by day 10.
