# Supplementary material for: AT2R Activation Improves Wound Healing in a Preclinical Mouse Model
Source: Biomedicines. 2024 Jun 3;12(6):1238. doi: 10.3390/biomedicines12061238 (PMC11200587; doi:10.3390/biomedicines12061238)
Supplement: Supplementary file 1 [file biomedicines-12-01238-s001.zip › biomedicines-2995371-supplementary.pdf]

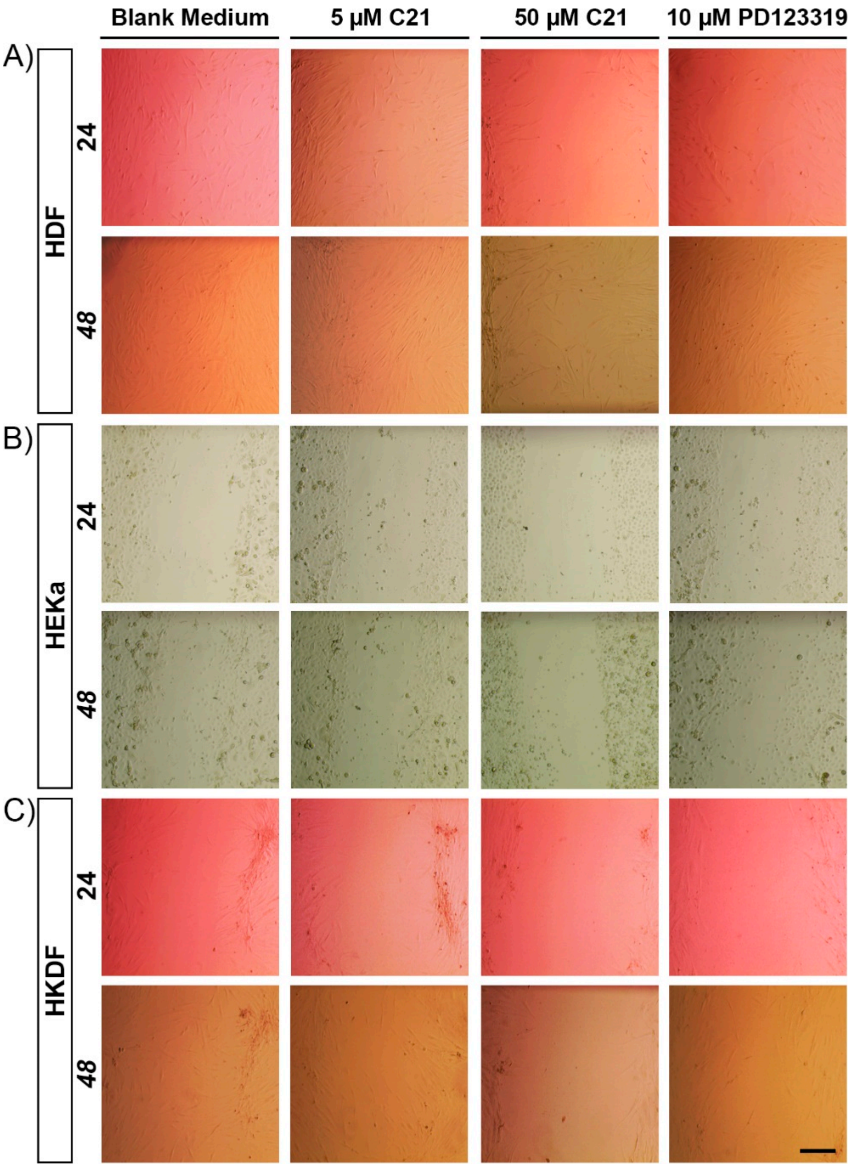

Figure S1. Scratch assays in human skin cell culture. Representative brightfield images acquired at 10x of scratched areas at 24 and 48 hours in A) human dermal fibroblasts (HDF), B) human keratinocytes (HEKa) C) human keloid dermal fibroblasts (HKDF) treated with 5 µM C21, 50 µM C21, 10 µM PD123319, or blank medium. Scale =250 µm.

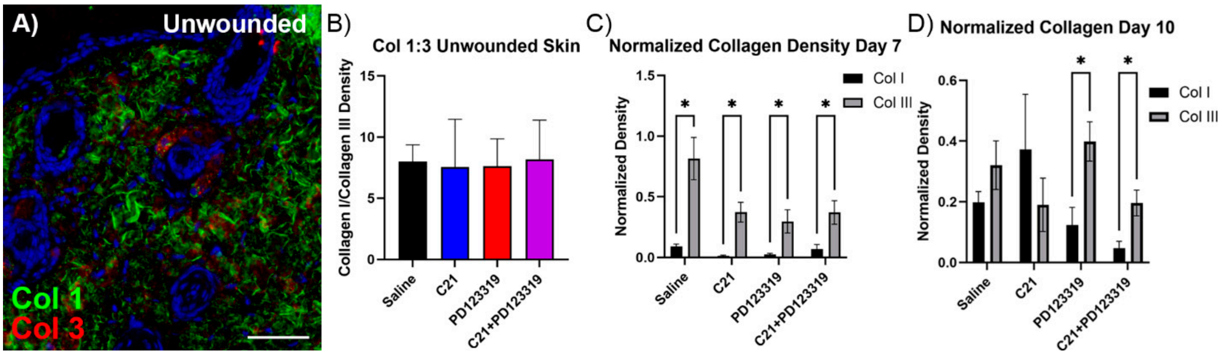

Figure S2. Collagen I and III densities in wounded and unwounded skin. A) Fluorescence image of unwounded skin at day 10 acquired at 20x and stained for collagen I (Col 1; green), collagen III (Col 3; red), and DAPI (blue). Scale bar = 100 μm. B) Ratio of collagen I:collagen III for unwounded skin from each treatment group at day 10 (mean±SEM), all comparisons ns. Normalized densities (mean±SEM) of collagen I and III for wounded skin in each treatment group at C) day 7 (*p<0.000001 saline Col I vs. Col III, *p=0.00016 C21 Col I vs. Col III, *p=0.00031 PD123319 Col I vs. Col III and *p=0.0148 combination Col I vs. Col III) and D) day 10 post-wounding (*p=0.0104 PD123319 Col I vs Col III and *p=0.0047 combination Col I vs Col III). Multiple Mann Whitney tests. Showing lesser densities of mature collagen type I in PD123319 and combination treated groups by day 10.
